# Association between immuno-nutritional biomarkers and mortality in hospitalized geriatric population

**DOI:** 10.3389/fimmu.2025.1692551

**Published:** 2025-11-20

**Authors:** Serena S Stephenson, Ganna Kravchenko, Anna Gawron-Skarbek, Tomasz Kostka, Bartłomiej K Sołtysik

**Affiliations:** Department of Geriatrics, Healthy Ageing Research Centre, Medical University of Lodz, Łódź, Poland

**Keywords:** prognostic nutritional index (PNI), immunonutritional biomarkers, aging, immune response, in-hospital mortality

## Abstract

**Objectives:**

This study aimed to identify the most sensitive immuno-nutritional and systemic inflammation biomarkers for predicting in-hospital all-cause mortality in older adults.

**Methods and material:**

A retrospective observational study was conducted in 2,067 hospitalized geriatric patients aged ≥60 years in the Department of Geriatrics, Lodz, Poland, from 2017 to 2024. Blood-based immuno-nutritional indices were calculated from routine laboratory tests at admission, including NLR (Neutrophil-to-Lymphocyte Ratio), LMR (Lymphocyte-to-Monocyte Ratio), PNI (Prognostic Nutritional Index), PLR (Platelet-to-Lymphocyte Ratio), LCR (Lymphocyte-to-C-Reactive Protein Ratio), DLR (D-dimer-to-Lymphocyte Ratio), MWR (Monocyte-to-White Blood Cell Ratio), SII (Systemic Immune-Inflammation Index), SIRI (Systemic Inflammation Response Index), CAR (C-Reactive Protein-to-Albumin Ratio), DAR (D-dimer to Albumin Ratio), PAR (Platelet-to-Albumin Ratio), NAR (Neutrophil-to-Albumin Ratio), PIV (Pan-Immune-Inflammation Value), C-Reactive Protein (CRP) and White Blood Cell (WBC) count. Differences between survivors and non-survivors were analyzed using Mann–Whitney U and Chi-square tests. Prognostic accuracy was assessed via Receiver Operating Characteristic (ROC) curve analysis, with statistical significance set at p ≤ 0.05. Additionally, multivariable logistic regression, calibration assessment, and 10-fold cross-validation were used to confirm the robustness and internal validity of prognostic models.

**Results:**

The mean age was 80.88 ± 8.33 years for men and 82.92 ± 7.72 years for women. Men had higher levels of inflammatory biomarkers (NLR, SIRI, CAR), while women exhibited better nutritional and immune profiles (higher PNI, LMR). Non-survivors of both sexes showed significantly higher NLR, PLR, DLR, SII, SIRI, CAR, DAR, PAR, NAR and PIV, and significantly lower levels of LMR, PNI, LCR and MWR compared to survivors (p < 0.001). The Prognostic Nutritional Index (PNI) demonstrated the highest predictive value for in-hospital mortality (AUC = 0.837; sensitivity = 0.88, specificity = 0.64), followed by CAR and LCR. Other indices, including DLR, DAR, and NAR, also showed significant but comparatively lower predictive accuracy. In multivariable analysis, age, PNI, LCR, and NAR remained independent predictors of mortality (AUC for final model = 0.852).

**Conclusion:**

This study highlights PNI as the most sensitive and reliable biomarker for predicting in-hospital mortality among older adults. These results support using PNI and inflammatory markers in clinical assessments to better identify high-risk geriatric patients and reduce mortality.

## 1 Introduction

Malnutrition and chronic inflammation are interlinked conditions that significantly impact mortality among older adults (1). Aging is associated with immunosenescence and a chronic low-grade inflammatory state known as “inflammaging,” both of which contribute to frailty, functional decline, and vulnerability to acute stressors (2). Over time, these changes can lead to serious health decline and increased risk of mortality. Consequently, accessible biomarkers that reflect both nutritional and inflammatory status are essential for risk stratification and individualized care in geriatric populations. In this context, numerous immuno-nutritional and systemic inflammation indices derived from routine blood tests have gained attention for their prognostic value in predicting mortality. These biomarkers are non-invasive, cost-effective, and easily obtainable, making them especially suitable for clinical application in individuals of advanced age (3).

The Neutrophil-to-Lymphocyte Ratio (NLR) is one of the most widely studied indices, reflecting both immune suppression and systemic inflammation. Elevated NLR levels have been associated with increased all-cause and disease-specific mortality in older adults with various conditions, including cardiovascular diseases, neoplasms, and infections (4). Similarly, the Platelet-to-Lymphocyte Ratio (PLR) and Lymphocyte-to-Monocyte Ratio (LMR) have shown significant associations with mortality risk in hospitalized older patients and those with chronic diseases (5). The Prognostic Nutritional Index (PNI), which combines serum albumin and lymphocyte count, serves as a composite indicator of both nutritional and immune status. This is supported by findings from a recent cross-sectional study, in which the PNI showed the strongest and most consistent associations with age, BMI, and prevalent chronic conditions such as atrial fibrillation, diabetes, and dementia in a hospitalized geriatric population (6). Low PNI values have been consistently linked to poor survival outcomes in geriatric patients, especially those with malignancies or undergoing surgery (5, 7).

Expanding on these core markers, newer indices such as the Lymphocyte-to-C-Reactive Protein Ratio (LCR), incorporating acute-phase reactants and enhancing sensitivity to systemic inflammation, have been proposed (8). These markers have shown prognostic relevance in elderly patients with infections, sepsis and cancer.

Thrombo-inflammatory markers are gaining attention like the D-dimer-to-Lymphocyte Ratio (DLR) (9) for its association with mortality in conditions involving coagulation abnormalities, including COVID-19 and venous thromboembolism, both of which disproportionately affect older adults. D-dimer-to-Albumin Ratio (DAR) (10) has recently surfaced as a promising biomarker reflecting the balance between coagulation activation and nutritional/inflammatory status in various clinical conditions (11). By combining these two parameters, DAR provides a composite index that may better capture the severity of underlying disease processes, particularly those involving inflammation and hypercoagulability (12).

Likewise, the Monocyte-to-White Blood Cell Ratio (MWR) has shown associations with immune suppression and mortality, particularly in frail and immunocompromised elderly patients (13).

More integrative indices such as the Systemic Immune-Inflammation Index (SII) (14) and the Systemic Inflammation Response Index (SIRI), have demonstrated strong prognostic utility in predicting long-term outcomes in aging populations with cancer (15), cardiovascular disease (16), and infections (17).

The C-Reactive Protein-to-Albumin Ratio (CAR) (18), which reflects the balance between pro-inflammatory and nutritional status, has emerged as a strong independent predictor of mortality across various geriatric cohorts, including patients with sepsis and chronic diseases (19–21). Additionally, the Neutrophil-to-Albumin Ratio (NAR) (22) and Platelet-to-Albumin Ratio (PAR) (23) further develop the capacity to integrate inflammatory burden with nutritional impact.

A particularly novel and comprehensive marker is the Pan-Immune-Inflammation Value (PIV) (24), which combines neutrophils, monocytes, platelets, and lymphocytes into a single index. PIV has demonstrated strong predictive value for mortality and treatment outcomes in older cancer patients, providing a more comprehensive view of immune dysregulation, systemic stress, and infection risk (17, 25).

Although numerous studies have explored the utility of various biomarkers in relation to specific clinical outcomes within the context of particular diseases or physiological stressors, there remains relative paucity of research directly comparing a broad spectrum of biomarkers under hospital conditions particularly regarding their predictive value for all-cause mortality. Therefore, the present study aims to investigate the prognostic significance of a comprehensive panel of immuno-nutritional and systemic inflammation biomarkers in relation to all-cause mortality among hospitalized geriatric patients.

## 2 Materials and methods

### 2.1 Study design

**Figure 1:**
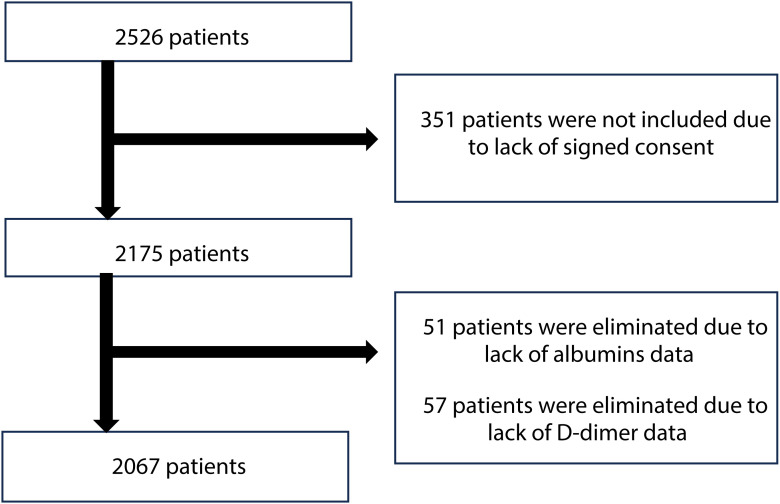
Flowchart of selecting the research group.

The study population was consisted of older adults, aged 60 years old and above, who were hospitalized in the Department of Geriatrics located in Lodz, Poland. Patients were recruited from January 2017 to December 2024. Between 2020 and 2022, the department was transformed to serve as a COVID-19 ward and patients admitted during this time were not included in the analysis. Participants were selected based on the following inclusion criteria: admission to the department, age 60 years or older, ability to communicate effectively, availability of complete data, and provision of informed signed consent. Individuals who were unable to communicate effectively or had significant cognitive impairment preventing obtaining informed consent (351 people) were not enrolled, which resulted in an initial study cohort of 2,175 patients (**Figure 1**). Additionally, 57 patients were excluded due to lack of D-dimer data. Furthermore, 51 patients were eliminated due to the inadequacy of albumins data. Patients with comorbidities, chronic as well as those with acute conditions, including infections, were included into the study. Following screening, 2,067 patients (619 men and 1,448 women) who met the inclusion criteria were enrolled in the analysis.

### 2.2 Data collection

Data collection for the study involved obtaining information on age and sex. Laboratory parameters, including a full blood count (white blood cell count, neutrophils, monocytes, lymphocytes, and platelets), were analyzed using the Sysmex XN 2000 analyzer (Kobe, Japan). Additionally, CRP level, albumin and D-dimer concentration were determined using the Beckman Coulter Dx700 AU analyzer (Brea, CA, USA). Measurements of body mass and height were taken with participants barefoot, facilitating the calculation of the Body Mass Index (BMI). All laboratory parameters were obtained upon patient admission.

Immunonutritional biomarkers were evaluated as follows:

NLR is determined by dividing the absolute neutrophil count by the absolute lymphocyte count in a blood sample (26). LMR is assessed by dividing the absolute lymphocyte count by the absolute monocyte count in a blood sample (27). The prognostic nutritional index (PNI) is calculated with the equation of PNI = 10×serum albumin (g/dL) + 0.005 × total lymphocyte count (per mm^3^) (28). The PLR was calculated as platelet count divided by absolute lymphocyte count (29).

LCR is computed by dividing the absolute lymphocyte count by the CRP [mg/L] in a blood sample (8). D-dimer to lymphocyte (DLR) is formulated by dividing the d-dimer concentration (mg/L) by the lymphocyte count (30). MWR is analyzed by dividing the absolute monocyte count by the total white blood cell count in a blood sample (31).

The Systemic Immune–Inflammatory Index (SII) represents a numeric value derived from peripheral blood counts, specifically the platelet count, neutrophil count, and lymphocyte count (32). It is assessed by multiplying the neutrophil count and platelet count, then dividing the lymphocyte count. The Systemic Inflammation Response Index (SIRI) is measured by multiplying the neutrophil count by the monocyte count, then dividing the result by the lymphocyte count (33). **Table 1** shows the formulas and unit conventions for commonly used inflammatory and nutritional biomarkers in older adults.

**Table 1:** Formulas and units for inflammatory and biomarkers.

Biomarker	Formula	Albumin unit used	Other units
NLR	Neutrophils ÷ Lymphocytes	–	both ×10^9^/L
LMR	Lymphocytes ÷ Monocytes	–	×10^9^/L
PNI	10 × Albumin (g/dL) + 0.005 × Lymphocytes (/mm^3^)	g/dL	Lymphocytes in cells/mm³
PLR	Platelets ÷ Lymphocytes	–	×10^9^/L
LCR	Lymphocytes (×10^9^/L) ÷ CRP (mg/L)	–	Lymphocytes in ×10^9^/L, CRP in mg/L
DLR	D-dimer (mg/L) ÷ Lymphocytes (×10^9^/L)	–	mg/L, ×10^9^/L
MWR	Monocytes ÷ WBC	–	×10^9^/L
SII	(Platelets × Neutrophils) ÷ Lymphocytes	–	×10^9^/L
SIRI	(Neutrophils x monocytes) ÷lymphocytes	–	×10^9^/L
CAR	CRP (mg/L) ÷ Alb (g/dL)	g/dL	CRP mg/L
DAR	D-dimer (mg/L) ÷ Alb (g/dL)	g/dL	D-dimer mg/L
PAR	Platelets (×10^9^/L) ÷ Alb (g/L)	g/L	Platelets ×10^9^/L
NAR	Neutrophils (×10^9^/L) ÷ Alb (g/L)	g/L	Neutrophils ×10^9^/L
PLR	Platelets ÷ Lymphocytes	–	×10^9^/L
PIV	(Neutrophils × Platelets × Monocytes) ÷ Lymphocytes	–	×10^9^/L

The CRP-to-albumin ratio (CAR) is evaluated by dividing CRP [mg/L] by albumin [g/dL] (34). DAR is measured by dividing D-dimer to albumin. The platelet to albumin ratio (PAR) is quantified by dividing the platelet count by the albumin concentration (g/L) (35). Neutrophil to albumin ratio (NAR) derived by dividing the neutrophil count by albumin level (36). Pan- Immune-Inflammation Value (PIV) is assessed by multiplying the neutrophil count, platelet count, monocyte count, and then dividing the result by the lymphocyte count (37). In addition to the composite immunonutritional indices, C-Reactive Protein (CRP) and White Blood Cell (WBC) count were also evaluated as individual inflammatory biomarkers for comparative purposes. Their values were extracted from the same admission blood tests and included in the predictive analysis (**Table 2**).

**Table 2:** Biomarker levels compared between survivors and non-survivors, with categorization by sex.

Biomarker	Gender	N=1913		N= 154		*p* value*
		Patients discharged		Patients deceased		
		Mean ± SD	Median (Quartiles)	Mean ± SD	Median (Quartiles)	
Age [years]		82.05 ± 7.95	83(77-88)	84.52 ± 7.42	87(81-91)	p< 0.001
BMI [kg/m^2^]		26.98 ± 7.69	25.95(22.89-29.72)	37.18 ± 27.94	25.63(22.96-32.62)	p=0.49
Body mass [kg]		68.09 ± 16.04	66.80(57-77)	66.95 ± 15.69	65(56-75)	p=0.55
NLR	Women	4.57 ± 6.56	3.06(2.06-4.79)	8.69 ± 7.93	6.45(3.86-11.00)	p< 0.001
	Men	4.87 ± 6.12	3.36(2.25-5.29)	9.74 ± 8.83	7.47(5.11-12.77)	p < 0.001
LMR	Women	2.89 ± 2.48	2.55(1.80-3.48)	2.14 ± 1.54	1.67(1.17-2.62)	p < 0.001
	Men	2.44 ± 1.38	2.23(1.54-3.12)	2.24 ± 3.14	1.34(1.10-1.98)	p < 0.001
PNI	Women	37.62 ± 5.96	38.60 (33.71-42.11)	28.91 ± 5.00	28.85 (25.06-32.60)	p < 0.001
	Men	36.75 ± 6.45	37.60 (32.30-41.81)	29.65 ± 6.82	31.80 (24.62-34.50)	p < 0.001
PLR	Women	188 ± 136	155(113-214)	233 ± 193	187(110-276)	p =0.56
	Men	172 ± 128	140(101-203)	255 ± 188	183(145-317)	p < 0.001
LCR	Women	0.78 ± 1.58	0.29(0.06-0.93)	0.15 ± 0.41	0.02(0.009-0.09)	p < 0.001
	Men	0.69 ± 1.24	0.20(0.03-0.74)	0.07 ± 0.17	0.01(0.006-0.06)	p < 0.001
DLR	Women	2.10 ± 6.66	0.69(0.34-1.56)	6.15 ± 14.6	2.46(1.10-4.37)	p < 0.001
	Men	2.87 ± 11.60	0.74(0.32-1.82)	8.35 ± 16.7	2.67(1.21-5.25)	p < 0.001
MWR	Women	0.10 ± 0.44	0.08(0.06-0.09)	0.07 ± 0.03	0.07(0.05-0.09)	p < 0.001
	Men	0.10 ± 0.34	0.08(0.07-0.10)	0.07 ± 0.04	0.07(0.04-0.09)	p =0.098
SII	Women	1153 ± 1665	724(456-1180)	2298 ± 3092	1407(700-2764)	p < 0.001
	Men	1106 ± 1729	677(416-1180)	2070 ± 2235	1407(808-2577)	p < 0.001
SIRI	Women	3.74 ± 15.04	1.77(1.12-3.26)	7.77 ± 10.88	4.61(2.33-8.54)	p < 0.001
	Men	4.29 ± 12.9	2.15(1.33-4.06)	8.29 ± 15.31	4.75(2.16-7.84)	p < 0.001
CAR	Women	9.06 ± 18.75	1.31(0.42-6.14)	30.35 ± 29.10	24.26(4.51-52.80)	p < 0.001
	Men	11.21 ± 20.32	1.87(0.51-11.78)	36.71 ± 35.43	33.21(6.50-50.86)	p < 0.001
DAR	Women	0.73 ± 1.98	0.27(0.14-0.58)	2.31 ± 4.48	0.90(0.49-2.14)	p < 0.001
	Men	0.90 ± 3.23	0.29(0.12-0.63)	2.34 ± 4.49	0.85(0.35-1.89)	p < 0.001
PAR	Women	6.80 ± 3.14	6.06(4.87-7.89)	8.91 ± 5.47	7.43(5.68-11.61)	p < 0.001
	Men	6.22 ± 3.20	5.50(4.26-7.17)	7.92 ± 4.98	6.27(4.45-9.90)	p =0.42
NAR	Women	0.16 ± 0.16	0.12(0.09-0.17)	0.34 ± 0.28	0.27(0.17-0.42)	p < 0.001
	Men	0.16 ± 0.11	0.13(0.09-0.19)	0.28 ± 0.17	0.23(0.15-0.33)	p < 0.001
PIV	Women	898 ± 2224	435(242-859)	2244 ± 4836	902(459-2528)	p < 0.001
	Men	1006 ± 2974	435(237-896)	1885 ± 4161	726(377-1842)	p < 0.001

BMI, Body Mass Index; NLR, Neutrophil-to-Lymphocyte Ratio; LMR, Lymphocyte-to-Monocyte Ratio; PNI, Prognostic Nutritional Index; PLR, Platelet-to-Lymphocyte Ratio; LCR, Lymphocyte-to-C-Reactive Protein Ratio;DLR, D-dimer- Lymphocyte Ratio; MWR, Monocyte-to-White Blood Cell Ratio; SII, Systemic Immune-Inflammation Index; SIRI, Systemic Inflammation Response Index; CAR, C-Reactive Protein-to-Albumin Ratio; DAR, D-dimer to Albumin Ratio; PAR, Platelet-to-Albumin Ratio; NAR, Neutrophil-to-Albumin Ratio; PIV, Pan-Immune-Inflammation Value.

*Adjusted for Bonferroni correction.

### 2.3 Statistical analysis

As several variables were not normally distributed, data were expressed both as mean ± standard deviation (SD) and as median (quartiles). Due to non-normal distribution and lack of homogeneity of variance, quantitative variables were compared using the Mann–Whitney U-test with Bonferroni correction.

Receiver operating characteristic (ROC) curves were used to assess the predictive capacity of immunonutritional biomarker–based indicators for mortality, and the area under the curve (AUC) was evaluated accordingly. No formal sample-size calculation was conducted, as this was a retrospective study including all eligible patients during the study period. With 2,067 patients and 154 events (in-hospital deaths), the sample size was sufficient for the planned univariable analyses, meeting commonly recommended events-per-variable criteria for prognostic studies (38).

A stepwise multivariable logistic regression model (binomial distribution, logit link) was fitted to estimate the association between predictors and mortality. Wald statistics and odds ratios (ORs) with 95% confidence intervals (CIs) were calculated. Predicted probabilities from the model were saved for subsequent discrimination and calibration analyses.

Model discrimination was evaluated using the area under the ROC curve (AUC) with standard error and 95% CI. The optimal classification threshold was determined using the Youden index, and sensitivity, specificity, accuracy, positive and negative predictive values (PPV and NPV), and likelihood ratios were reported at this cut-off.

Calibration was assessed using three complementary methods: the Hosmer–Lemeshow goodness-of-fit test (10 deciles), a calibration model regressing the outcome on the logit of predicted probabilities to obtain calibration intercept and slope, and decile-wise calibration plots comparing observed vs. predicted risk.

To address potential overfitting and evaluate internal validity, we performed 10-fold stratified cross-validation. The dataset was randomly divided into 10 stratified folds. For each fold, the model was trained on nine folds and tested on the held-out fold. Out-of-fold predictions were aggregated to compute global performance metrics, including cross-validated AUC and Brier score, as well as fold-wise AUCs to assess model stability. Statistical significance was set at *p* ≤ 0.05. Statistical analysis was performed using Statistica 13.1 (StatSoft Sp. z o. o., Kraków, Poland).

## 3 Results

### 3.1 Sex-based differences in anthropometric and immunonutritional profiles.

**Table 3** shows anthropometry and immunonutritional markers in the group of 2,067 patients by sex. Women were significantly older than men (82.92 ± 7.72 vs. 80.88 ± 8.33 years, p < 0.001). BMI was similar between sex groups (27.0 ± 9.3 vs. 27.2 ± 9.2 kg/m², p = 0.63), but men had significantly higher body mass (76.0 ± 15.4 vs. 64.0 ± 15.1 kg, p < 0.001).

**Table 3:** Characteristics of the subjects according to sex.

Variable	Women n= 1448 Mean ± SD (Median and quartiles)	Men n= 619 Mean ± SD (Median and quartiles)	*p*-value
Age [years]	82.92 ± 7.72 84 (78–89)	80.88 ± 8.33 82 (74–87)	p < 0.001
BMI [kg/m^2^]	27.36 ± 9.31 26(23-30)	27.21 ± 9.20 26(23-29)	p=0.63
Body mass [kg]	64.79 ± 15.13 63(55-73)	75.70 ± 15.47 73(66-84)	p < 0.001
NLR	4.86 ± 6.74 3.17(2.12-5.15)	5.29 ± 6.53 3.55(2.42-5.75)	p < 0.001
LMR	2.84 ± 2.43 2.48(1.71-3.42)	2.42 ± 1.61 2.17(1.45-3.06)	p < 0.001
PNI	37.02 ± 6.30 37.90(33.00-41.81)	36.12 ± 6.77 37.00 (31.80-41.51)	p=0.014
PLR	192.19 ± 143.69 156.62(113.72-219.75)	179.66 ± 136.78 145.30(103.79-209.70)	p=0.003
LCR	0.75 ± 1.53 0.25(0.04-0.86)	0.64 ± 1.20 0.16(0.02-0.66)	p < 0.001
DLR	2.38 ± 7.54 0.73(0.36-1.80)	3.34 ± 12.21 0.83(0.34-2.13)	p=0.30
MWR	0.11 ± 0.43 0.08(0.06-0.09)	0.10 ± 0.32 0.08(0.07-0.10)	p < 0.001
SII	1235.96 ± 1825.17 747.56(464.16-1270.64)	1189.49 ± 1796.32 706.79(436.03-1263.43)	p=0.39
SIRI	4.02 ± 14.82 1.85(1.14-3.51)	4.64 ± 13.16 2.24(1.37-4.40)	p < 0.001
CAR	10.53 ± 20.34 1.49(0.45-8.13)	13.47 ± 23.16 2.24(0.56-16.21)	p=0.002
DAR	0.84 ± 2.28 0.29(0.15-0.65)	1.03 ± 3.37 0.31(0.13-0.73)	p=0.54
PAR	6.96 ± 3.40 6.13(4.89-8.03)	6.37 ± 3.42 5.55(4.26-7.32)	p < 0.001
NAR	0.17 ± 0.18 0.12(0.09-0.19)	0.17 ± 0.12 0.13(0.09-0.20)	p=0.012
PIV	993.71 ± 2514.74 458.87(245.98-912.71)	1083.18 ± 3099.01 458.28(244.45-971.78)	p=0.59
Discharged; n (%)	1348(93.02%)	565(91.12%)	
Death; n (%)	100 (6.98%)	54(8.88%)	p=0.51

BMI, Body Mass Index; NLR, Neutrophil-to-Lymphocyte Ratio; LMR, Lymphocyte-to-Monocyte Ratio; PNI, Prognostic Nutritional Index; PLR, Platelet-to-Lymphocyte Ratio; LCR, Lymphocyte-to-C-Reactive Protein Ratio; DLR, D dimer- Lymphocyte Ratio; MWR, Monocyte-to-White Blood Cell Ratio; SII, Systemic Immune-Inflammation Index; SIRI, Systemic Inflammation Response Index; CAR,C-Reactive Protein-to-Albumin Ratio; DAR, D-dimer to Albumin Ratio; PAR, Platelet-to-Albumin Ratio; NAR, Neutrophil-to-Albumin Ratio; PIV, Pan-Immune-Inflammation Value. Data are expressed both as the mean ± SD and median (25–75% of quartiles).

Several inflammatory and nutritional markers differed by sex. Men had higher NLR, SIRI, and CAR values, indicating greater systemic inflammation (p < 0.01 for all). Women had higher LMR, PNI, PLR, LCR, MWR, and PAR, suggesting better immune and nutritional status (p < 0.05 for all). No significant differences were found in SII, NAR, DLR, DAR, or PIV. In-hospital mortality did not differ significantly between sexes (7.0% in women vs. 8.9% in men, p = 0.51).

### 3.2 Predictive accuracy of immunonutritional markers for mortality risk.

In **Table 2**, we present differences in immunonutritional marker levels between discharged and deceased patients, with sex stratification. Most markers were strongly associated with in-hospital mortality. In both women and men, those who died had significantly higher levels of NLR, DLR, SII, SIRI, CAR, DAR, NAR, and PIV, and significantly lower levels of LMR, PNI, and LCR. While MWR was significantly lower in deceased women (p < 0.001), the difference was not statistically significant in men (p = 0.098). Additionally, PLR was not significant in women (p = 0.56), and PAR was not significant in men (p = 0.42).

### 3.3 Classification of markers based on mortality.

**Figure 2:**
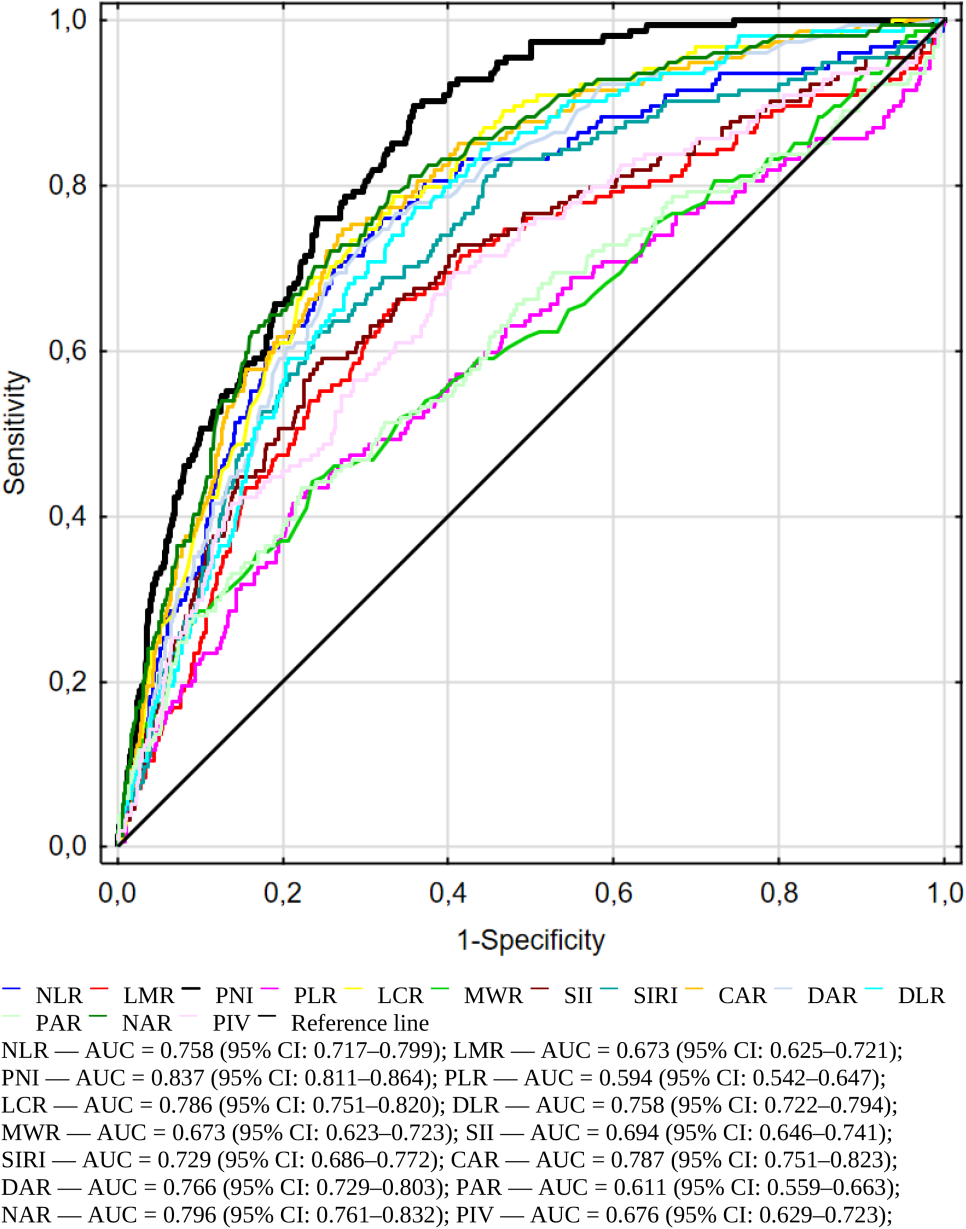
Receiver operating characteristic (ROC) curves illustrating the predictive accuracy of immuno-nutritional and inflammatory biomarkers for in-hospital mortality among geriatric patients.

In the next step, we evaluated the ability of several inflammatory and immune-nutritional markers to predict mortality using an ROC curve analysis (**Figure 2**). AUC, cut-off values, sensitivity, specificity, and Youden index are shown in **Table 4**. All markers were statistically significant (p < 0.001), supporting their relevance in mortality risk assessment. Among all markers, the Prognostic Nutritional Index (PNI) had the best performance, with an AUC of 0.837 (95% CI: 0.811–0.864). It showed high sensitivity (0.88) and moderate specificity (0.64) at the cut-off value of 35.70, making it the strongest predictor of mortality among all evaluated parameters. Furthermore, **Table 4** contains the data for C-Reactive Protein and White Blood Cells (not presented in **Figure 2**). PNI demonstrates higher sensitivity compared to two well-known and widely used inflammatory markers.

**Table 4:** Biomarkers in mortality prediction based on ROC analysis.

Biomarkers	AUC	L-U 95%	Youden index	Cut-off point	Sensitivity	Specificity	p value
NLR	0.758	0.717-0.799	0.44	4.18	0.76	0.68	p < 0.001
LMR	0.673	0.625-0.721	0.32	1.97	0.65	0.66	p < 0.001
PNI	**0.837**	**0.811-0.864**	**0.54**	**35.70**	**0.88**	**0.64**	**p < 0.001**
PLR	0.594	0.542-0.647	0.21	219.17	0.43	0.77	p < 0.001
LCR	0.786	0.751-0.82	0.45	0.10	0.78	0.66	p < 0.001
DLR	0.758	0.722-0.794	0.41	1.09	0.76	0.64	p < 0.001
MWR	0.673	0.623-0.723	0.32	0.81	0.50	0.81	p < 0.001
SII	0.694	0.646-0.741	0.34	1192.31	0.59	0.75	p < 0.001
SIRI	0.729	0.686-0.772	0.38	3.54	0.62	0.75	p < 0.001
CAR	0.787	0.751-0.823	0.47	5.97	0.74	0.72	p < 0.001
DAR	0.766	0.729-0.803	0.43	0.52	0.72	0.71	p < 0.001
PAR	0.611	0.559-0.663	0.21	7.99	0.43	0.77	p < 0.001
NAR	0.796	0.761-0.832	0.46	0.16	0.77	0.68	p < 0.001
PIV	0.676	0.629-0.723	0.29	540.58	0.69	0.59	p < 0.001
CRP	0.773	0.736-0.810	0.45	23.60	0.70	0.74	p < 0.001
WBC	0.695	0.649-0.742	0.31	10.00	0.50	0.81	p < 0.001

AUC, Area Under the Receiver Operating Characteristic Curve; L-U 95% – Lower–Upper 95% Confidence Interval; NLR, Neutrophil-to-Lymphocyte Ratio; LMR, Lymphocyte-to-Monocyte Ratio; PNI, Prognostic Nutritional Index; PLR, Platelet-to-Lymphocyte Ratio; LCR, Lymphocyte-to-C-Reactive Protein Ratio; DLR, D dimer- Lymphocyte Ratio; MWR, Monocyte-to-White Blood Cell Ratio; SII, Systemic Immune-Inflammation Index; SIRI, Systemic Inflammation Response Index; CAR,C-Reactive Protein-to-Albumin Ratio; DAR, D-dimer to Albumin Ratio; PAR, Platelet-to-Albumin Ratio; NAR, Neutrophil-to-Albumin Ratio; PIV, Pan-Immune-Inflammation Value; CRP, C-Reactive protein; WBC, White blood cells.

Bold values indicate the biomarker with the highest AUC (best discriminative performance).

**Figure 3:**
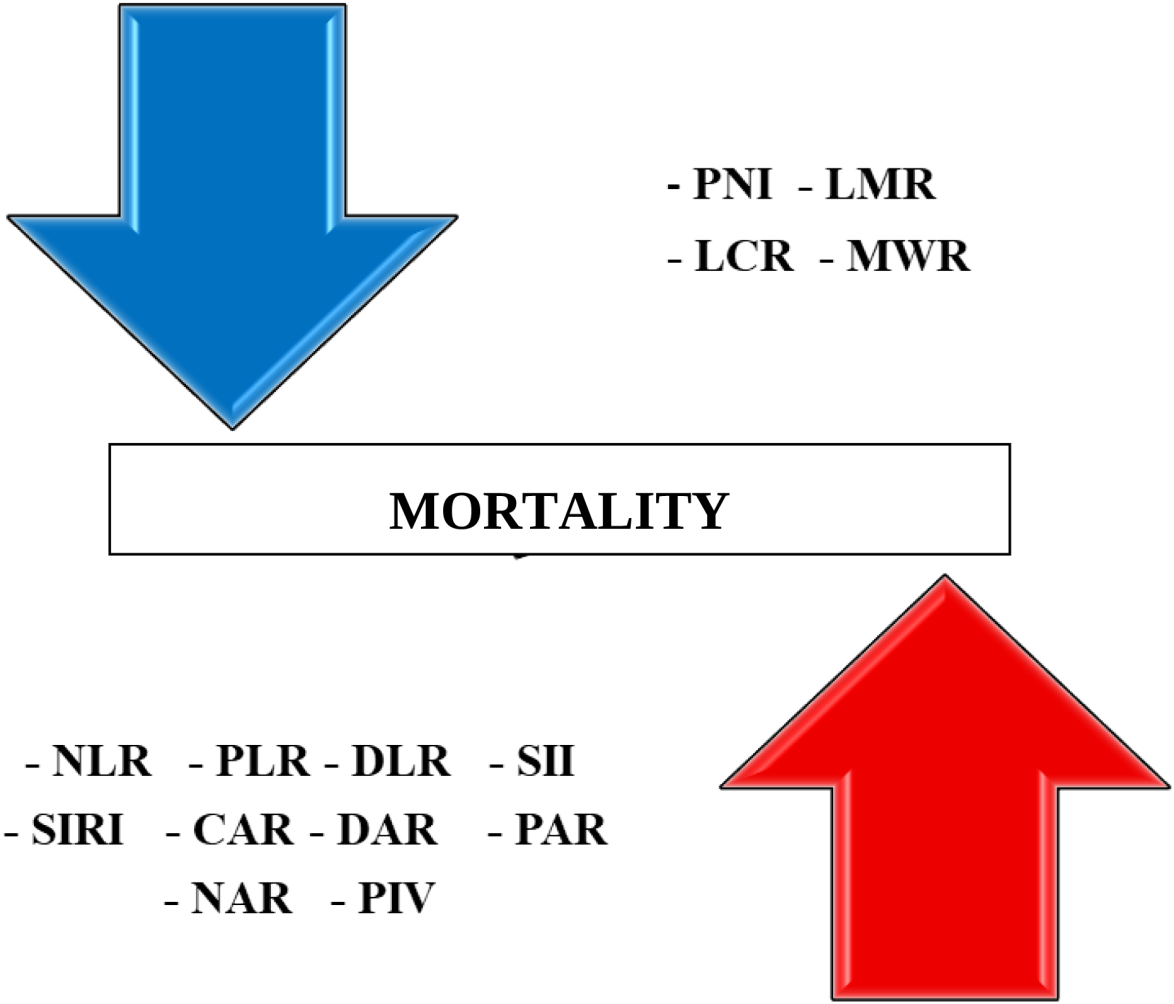
Summary panel of immuno-nutritional biomarkers associated with increased (lower and upward arrow) and decreased (upper and downward) risk of in-hospital mortality in older adults.

**Figure 3** distinguishes immunonutritional markers into two groups – those associated with increased and those with decreased risk of death during hospitalization. On the upper side, the downward arrow represents biomarkers associated with a decreased risk of death. These include PNI, LMR, LCR, and MWR. These biomarkers indicate better nutritional and immune status, such as higher lymphocyte counts or higher albumin levels, and were significantly higher in survivors. On the lower side, the upward arrow represents biomarkers associated with an increased risk of death during hospitalization. These include NLR, PLR, DLR, SII, SIRI, CAR, DAR, PAR, NAR, and PIV. These indices reflect elevated systemic inflammation, coagulopathy, or malnutrition, all of which contribute to worse clinical outcomes in geriatric patients. Their higher values were consistently observed in non-survivors (p < 0.001 for all).

### 3.4 Multivariable logistic regression analysis

To assess whether selected immuno-nutritional biomarkers remained independently associated with in-hospital mortality after accounting for confounders, stepwise multivariable logistic regression analysis was performed including age, PNI, NAR and LCR as (the strongest in univariate analysis) predictors (n = 2067; 154 deaths). The model showed good overall fit (Wald χ² = 247.16, p < 0.0001). As shown in **Table 5**, age (p < 0.001), PNI (p < 0.0001), NAR (p = 0.028), and LCR (p = 0.019) were significant independent predictors of in-hospital mortality. Higher PNI and LCR were associated with lower mortality risk, while lower NAR was also associated with reduced mortality. These findings confirm the independent prognostic value of key immuno-nutritional biomarkers beyond age effects. Corresponding odds ratios (95% CI) were: age 1.056 (1.031–1.081), PNI 0.872 (0.841–0.904), LCR 0.486 (0.265–0.891), and NAR 2.415 (1.101–5.298).

**Table 5:** Multivariable logistic regression analysis for in-hospital mortality.

Predictor	Wald χ²	p-value	OR	95% CI (lower–upper)
Intercept	4.44	0.035	—	—
Age [year]	20.12	<0.0001	1.056	1.031 – 1.081
PNI	54.96	<0.0001	0.872	0.841 – 0.904
LCR	5.45	0.0195	0.486	0.265 – 0.891
NAR	4.84	0.0279	2.415	1.101 – 5.298

### 3.5 Discrimination, calibration, internal validation

In the next steps we performed the set of controlling calculations. The multivariate model demonstrated good discrimination, with an AUC of 0.852 (SE 0.014; 95% CI 0.826–0.879) on the full dataset. The optimal classification threshold determined by the Youden index was 0.068, at which sensitivity was 0.838, specificity 0.715, accuracy 0.724, PPV 0.191, NPV 0.982, Positive Likelihood Ratio (LR+) 2.94, and Negative Likelihood Ratio (LR–) 0.55. At a conventional threshold of 0.50, very few observations were classified as positive (TP = 9, FP = 15, FN = 145, TN = 1897), resulting in high specificity (0.992) but low sensitivity (0.058) and an odds ratio of 7.85 (log OR = 2.06). In several smaller subgroups, no positive predictions occurred at this threshold, yielding degenerate 2×2 tables and infinite odds ratios, which reflects the skewed distribution of predicted probabilities rather than model misfit.

**Figure 4:**
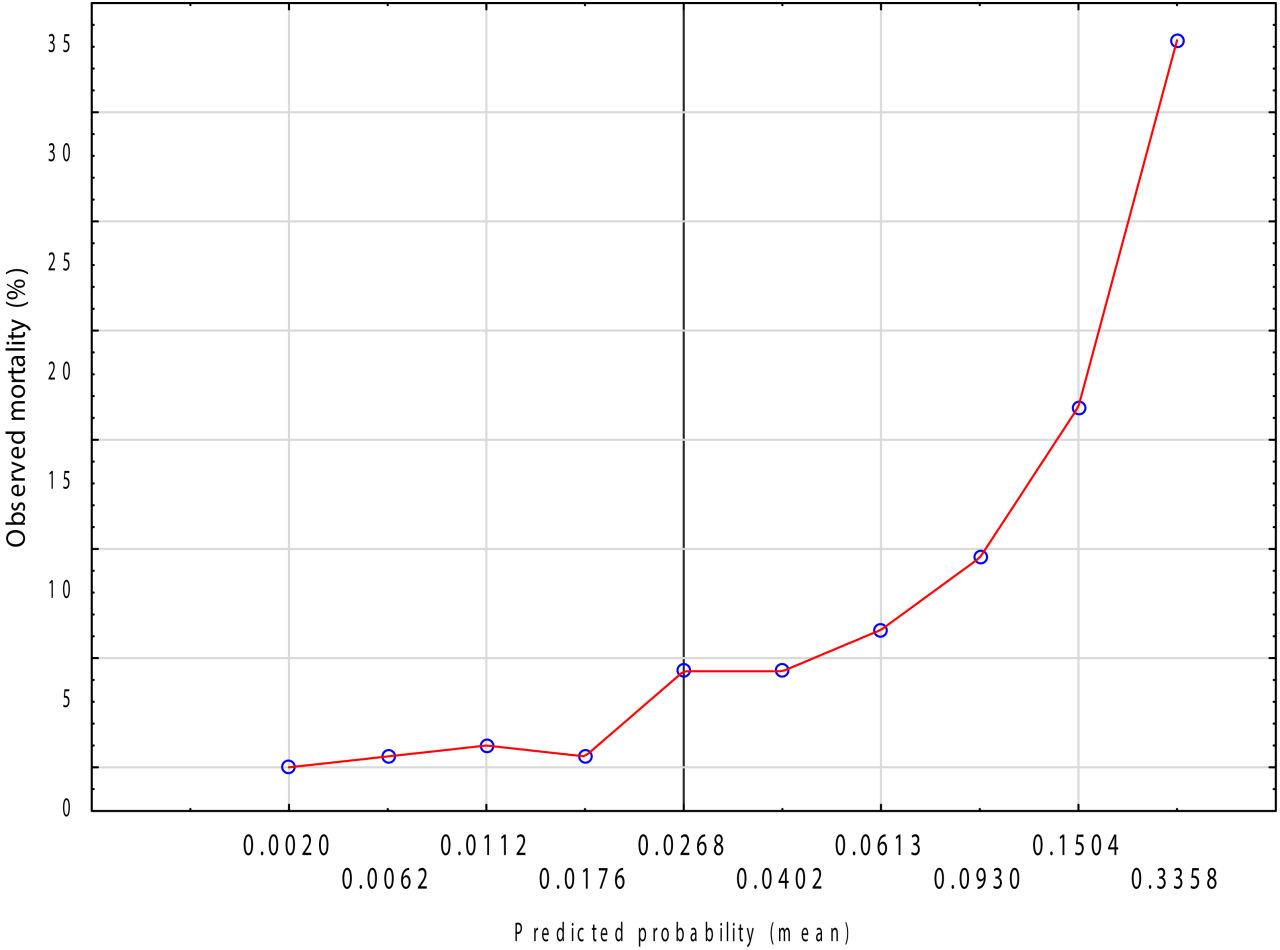
Calibration plot of the logistic regression model for in-hospital mortality.Observed vs. predicted probabilities across deciles of risk (LOWESS smoothed).

The Hosmer–Lemeshow goodness-of-fit test indicated no evidence of lack of fit (χ² = 4.868, *p* = 0.772). The calibration intercept was approximately 0, and the slope was close to 1, indicating no systematic under- or overestimation. Calibration plot across deciles of predicted risk demonstrated good agreement between observed and predicted event rates throughout the risk spectrum (**Figure 4**).

To assess internal validity and potential overfitting, we performed 10-fold stratified cross-validation. Fold-wise AUC values ranged from 0.805 to 0.902, indicating stable model performance across partitions. When out-of-fold predictions were aggregated, the cross-validated AUC was 0.887 (SE 0.012; 95% CI 0.864–0.910) with a Brier score of 0.053, supporting good discrimination and overall calibration after internal validation.

## 4 Discussion

To the best of our knowledge, this paper presents the first extensive and unique comparison of immunonutritional morphology derived biomarkers in hospitalized geriatric population. It is also the first to systematically evaluate the predictive accuracy of these biomarkers for in-hospital mortality, rather than focusing solely on disease presence or progression, by assessing a broad panel of markers.

### 4.1 Prognostic value of NLR and LMR

NLR and LMR are established markers of systemic inflammation and immune function, derived from standard blood count. In our study, both, elevated NLR and reduced LMR were strongly associated with increased in-hospital mortality among geriatric patients, regardless of gender (**Table 2**). By combining neutrophil and lymphocyte counts, the NLR provides a clearer picture of systemic inflammation, with elevated levels consistently associated with higher in-hospital mortality among older adults. In our study, NLR showed a strong ability to predict in-hospital mortality (AUC = 0.758, p < 0.001) (**Table 4**). A significantly lower LMR was consistently observed among non-survivors of both sexes, suggesting that a lower lymphocyte-to-monocyte ratio may reflect impaired adaptive immune response and enhanced innate inflammatory activity, both of which appear to contribute to elevated in-hospital mortality risk in older adults. A recent study in older adults admitted to emergency departments found that higher NLR values at admission were significantly linked to in-hospital mortality, even after adjustment for confounders (39). Similarly, lower LMR values have been linked to increased mortality in conditions such as cardiogenic shock and aortic dissection, supporting its role as a negative prognostic factor (40). This suggests that a higher NLR and lower LMR may reflect an overactive inflammatory response combined with weakened adaptive immunity, both of which are associated with increased in-hospital mortality in older adults.

### 4.2 PNI as the strongest predictor of in-hospital mortality

In presented study, PNI demonstrated the strongest predictive power for in-hospital mortality, with an area under the ROC curve (AUC) of 0.837, alongside high sensitivity (88%) and moderate specificity (64%) (**Table 4**). This underscores the critical role of nutritional and immune status in outcomes and aligns with previous studies on the prognostic value of PNI. Lower PNI values in deceased patients further reflect compromised nutritional reserves and impaired immunity, which may exacerbate vulnerability to adverse outcomes. This aligns with several studies, for instance a study on patients undergoing hemodialysis found that higher PNI quartiles were associated with lower mortality rates, with PNI demonstrating a better predictive ability than serum albumin or total lymphocyte counts alone. The study reported sensitivity of 88%, and specificity of 64% at a cut-off of 39.5 (41), similar to our study. Likewise, studies involving patients with acute ischemic stroke have shown that reduced PNI levels are strongly linked to increased 30-day mortality, highlighting the crucial role of nutritional and immune status in determining stroke prognosis (42). Although PNI demonstrated excellent sensitivity, its moderate specificity (0.64) indicates that it may not be sufficient as a standalone clinical tool. Rather, PNI should be considered as a screening marker, to be used in conjunction with other clinical assessments and biomarkers to improve overall prognostic accuracy in geriatric patients. In our study, the optimal classification threshold determined by the Youden index provided a balanced trade-off between sensitivity and specificity, and the robustness of the model was confirmed through 10-fold stratified cross-validation (overall cross-validated AUC = 0.887, Brier score = 0.053), supporting the reliability of the predictive performance. Clinically, this suggests that PNI may serve as a practical, inexpensive, and readily available marker to help identify older patients at higher risk of in-hospital mortality, enabling early nutritional and medical interventions that could potentially improve outcomes.

### 4.3 Prognostic implications of platelet, lymphocyte, CRP, and D-dimer-based ratios

PLR, a composite marker reflecting immune suppression, was significantly elevated in patients who died during hospitalization. However, its AUC in ROC analysis (0.594) suggests limited discriminative ability when used alone, particularly compared to other markers, for instance the prognostic nutritional index (PNI) or LCR (**Table 4**). Also, the study by Zhai et al. involving 5,577 cardiac intensive care unit patients found that higher PLR quartiles were associated with increased in-hospital mortality, with the highest quartile exhibiting a mortality rate of 13.9% compared to 8.3% in the lowest quartile (p < 0.001) (43). LCR, reflecting the balance between immunity (lymphocyte count) and systemic inflammation (CRP), demonstrated a stronger association with in-hospital mortality than PLR. In ROC analysis, LCR had a relatively high AUC of 0.786, indicating good prognostic performance. Similarly, the closely related marker CLR (CRP-to-lymphocyte ratio), which inversely mirrors LCR, has also shown prognostic value. In a study of older NSTEMI patients managed non-invasively, a higher CLR (lower LCR) was associated with an increased risk of in-hospital cardiac death (44). Additionally, among older patients with COVID-19, elevated CLR correlated with greater disease severity and higher in-hospital mortality [43]. These findings are supported by previous research demonstrating that lower LCR values are independently associated with higher mortality in patients with sepsis (16), heart failure (8), and advanced cancer (45). Next promising parameter is DLR, which in our study achieved an AUC of 0.76 (**Table 4**). This indicates on relatively high association with in-hospital mortality in geriatric population. In a cohort study of 1123 older septic patients, elevated DLR was associated with increased hospital mortality, and each 1-SD increase was folding 10% higher risk (46). Furthermore, subjects with highest DLR quartile had significantly higher cumulative mortality when compared with those with the lowest quartile (46). The study of over 10 thousand Spanish Covid-19 patients revealed that elevated DLR predicted in-hospital death with adjusted OR 2.12 and AUC 0.69 for mortality discrimination (47). What is important, DLR had better accuracy over sole D-Dimer or lymphocyte count in Covid-19 mortality models (47). In another Covid-19 related mortality study, DLR presented AUC = 0.92 for mortality prediction (9). To sum up, DLR integrates coagulation, inflammation and immune response, and shows independent predictive value for in−hospital mortality among older and critically ill population.

### 4.4 Clinical relevance of MWR in mortality stratification

MWR, representing the ratio of monocytes to total white blood cells and serving as an indicator of systemic inflammation (13), was significantly lower in non-survivors, though it demonstrated only moderate predictive accuracy for mortality (AUC = 0.673) (**Table 4**). The decreased MWR in deceased patients suggests that it may reflect heightened systemic inflammation, contributing to poor outcomes.

### 4.5 Immune-inflammatory indices SII and SIRI and their association with mortality outcomes

Next two indices, SII and SIRI, were increased in a group of deceased patients. SII, derived from platelet, neutrophil, and lymphocyte counts, serves as a comprehensive indicator of systemic inflammatory status. In our cohort, SII values were significantly higher in non-survivors compared to those who survived, in both men and women (**Table 2**). Moreover, ROC analysis demonstrated a moderate predictive value of SII for in-hospital mortality (AUC: 0.694), with a sensitivity of 69.5% and specificity of 59.3% at the optimal cut-off point of 1192.31 (**Table 4**). Furthermore, evidence from a large meta-analysis of 16 studies involving 10,007 hospitalized COVID-19 patients found that a high SII at admission was significantly linked to increase in-hospital mortality (Risk Ratio: 2.41, 95% CI: 1.78–3.24) (48). Similarly, a study of 267 Intensive Care Unit (ICU) patients with sepsis demonstrated that SII was an independent predictor of in-hospital mortality, with an adjusted odds ratio of 1.51 (95% CI: 1.24–1.84) (49). SIRI is an emerging biomarker that integrates neutrophil, monocyte, and lymphocyte counts to provide a comprehensive assessment of immune system activity. In our population, SIRI was significantly elevated in deceased patients, independent of sex. ROC curve analysis showed an AUC of 0.729, providing a sensitivity of 62% and specificity of 75% (**Table 4**). These results suggest that SIRI can serve as a moderately strong predictor of mortality. A 10-year retrospective cohort study investigated the association between SIRI and mortality risk in elderly patients with hip fractures. A study found that higher SIRI levels were significantly associated with increased 30-day and 1-year mortality rates (50).

### 4.6 Prognostic significance of CAR and DAR in mortality

Both male and female patients, who died had significantly elevated CAR and DAR values compared to survivors (p < 0.001) (**Table 2**). The prognostic performance of CAR was robust, with an AUC of 0.787, sensitivity of 0.74, and specificity of 0.72, underscoring its relevance as an immunonutritional marker linked to mortality risk (**Table 4**). CAR has also emerged as a significant prognostic marker in COVID-19 patients. A systematic review and meta-analysis demonstrated that elevated CAR level was associated with increased mortality and adverse clinical outcomes, underscoring its potential utility in clinical settings (51). Likewise, recent studies have demonstrated that a higher DAR was significantly associated with increased mortality risk. For example, Xiao et al. conducted a study involving 1,993 patients with COVID-19 and found that elevated DAR levels were predictive of severe illness and mortality (10). Similarly, results were obtained in the study that identified risk factors for mortality in COVID-19 patients admitted to intensive care units (52). Their findings highlighted that elevated D-dimer levels and decreased albumin concentrations were significantly associated with increased mortality risk.

### 4.7 Prognostic value of neutrophil-albumin and platelet-albumin ratios in mortality risk

Both the NAR and PAR combine counts of inflammatory cells (neutrophils or platelets) with albumin concentration, a well-known marker of nutritional and inflammatory status. In current study, NAR was significantly higher in patients who died compared to survivors in both sexes (**Table 2**), indicating that increased neutrophil-mediated inflammation combined with hypoalbuminemia is strongly associated with mortality risk. This study supports previous findings that NAR is a reliable prognostic marker, with ROC curve analysis showing that NAR predicts 90-day mortality in patients with cardiogenic shock more effectively than neutrophil percentage or serum albumin levels alone (53). In our population, PAR demonstrated statistically significant predictive value for mortality (p < 0.001), though its AUC of 0.611 suggests moderate discriminative ability (**Table 4**). This performance, while lower than other markers such as the PNI or NAR, still indicates that PAR captures a relevant aspect of the inflammatory-nutritional axis reflecting mortality risk. A study examining the association between Platelet-Albumin-Bilirubin (PALBI) grade, which incorporates PAR and mortality in patients with acute respiratory distress syndrome (ARDS) has confirmed that higher PALBI, and thus elevated PAR values, were significantly associated with increased risk of mortality (54).

### 4.8 Prognostic significance of pan-immune-inflammation value (PIV) in mortality risk

In this study, PIV was significantly higher in patients who died during hospitalization, indicating their potential as prognostic biomarkers for mortality. PIV, integrating counts of multiple immune and inflammatory cells, was also significantly elevated in non-survivors, but its predictive ability was more substantial (AUC = 0.676) (**Table 4**). Similarly, a study by Bo Wu et al. demonstrated that higher PIV levels were significantly associated with long-term all-cause mortality in patients with hypertension (55). The hazard ratio for all-cause mortality was 1.37 (95% CI: 1.20–1.55), indicating a strong association between elevated PIV and increased mortality risk. Recent findings in patients with sepsis-associated acute kidney injury showed that higher PIV quartiles were linked to increased 30-day and 1-year mortality, supporting its prognostic utility (56).

## 5 Limitations of the study

Many referenced studies focus on disease-specific populations, such as COVID-19, sepsis, or cancer. Our study involves a broad geriatric hospital population, enhancing the generalizability of these biomarkers for prognostic use.

Despite the strengths of this large retrospective cohort study, there are a number of limitations that must be considered. First, the observational nature of the study limits the ability to suggest causality between biomarker levels and mortality outcomes. While associations were consistent and statistically significant, potential confounding variables such as underlying comorbidities, the severity of illness, specific diagnoses and therapeutic interventions were not included in the analysis. These factors could independently influence inflammatory and nutritional status, and therefore the biomarker values. Second, this study utilized biomarker levels obtained only at the time of hospital admission. Although these values provide an early estimate of prognosis, they may not reflect dynamic changes during hospitalization that could better capture developing patient’s outcomes. Third, differences in healthcare systems, nutritional habits, and ethnicity may limit generalization. Additionally, only baseline biomarker values obtained at admission were analyzed in this study. However, repeated or serial measurements throughout hospitalization could offer stronger predictive insights by capturing the evolving clinical and physiological status of patients. Future prospective studies should directly assess the added prognostic value of these biomarkers alongside established clinical instruments.

## 6 Conclusions

This study underscores the prognostic relevance of immunonutritional and inflammatory biomarkers in predicting in-hospital mortality. Among the evaluated indices, the Prognostic Nutritional Index (PNI) emerged as the most powerful predictor, demonstrating excellent discriminative ability (AUC 0.837) with high sensitivity nd moderate specificity. These findings highlight the significant interaction between nutritional status and immune health in determining clinical outcomes, particularly in hospitalized geriatric populations. Lower PNI values in non-survivors reflect a state of malnutrition and immunosuppression, which may exacerbate vulnerability to systemic complications and poor recovery. PNI could be used as a simple, early screening tool at hospital admission to help identify very old patients at increased risk of adverse outcomes. This information could guide targeted nutritional interventions, such as early dietitian referral or individualized nutritional support, and prompt early comprehensive geriatric assessment. Additionally, integrating PNI into routine admission panels could support multidisciplinary decision making and prioritization of preventive measures in high-risk patients.

## Data Availability

The data analyzed in this study is subject to the following licenses/restrictions: The statistical data used to support presented findings may be obtained by sending a request to the corresponding author. Requests to access these datasets should be directed to Bartłomiej K. Sołtysik, bartlomiej.soltysik@umed.lodz.pl.
